# Supplementary material for: Workshop-based participatory research design through the lens of a culture-centered approach (CCA)
Source: PLoS One. 2025 May 7;20(5):e0322785. doi: 10.1371/journal.pone.0322785 (PMC12057982; doi:10.1371/journal.pone.0322785)
Supplement: Attachment — Submitted filename: Response .docx [file pone.0322785.s002.docx]

| Reviewer | Comment | Response |
| --- | --- | --- |
| Reviewer | • The abstract could mention the broader implications of the study. How can these findings be applied beyond just the NICU setting? • The abstract needs more detail about the impact of the findings. Be specific about how the proposed changes in communication infrastructure will improve NICU practices. | Addressed please see the abstract |
|  | • The introduction could provide more context about the existing literature on health communication in the NICU. It's important to clearly establish why the focus on nurses’ voices is necessary and how it adds to current research. • The introduction could benefit from a more concise description of the research problem. Some sections are a bit repetitive, especially when talking about the importance of nurses’ involvement. Streamlining this could make the introduction more focused. | Addressed throughout the 2introduction |
|  | • The introduction should include a clear research question or hypothesis. While the purpose is mentioned, framing it as a specific research question or problem to address will help readers follow the study’s goals more clearly. | Addressed please see the last paragraph |
|  | • The role of health communication in improving outcomes for both parents and newborns is mentioned, but it could be expanded. How does improving communication between nurses and parents directly affect patient outcomes or experiences in the NICU? | Addressed please see the first paragraph |
|  | • The introduction might be improved by framing the study in a broader context. How does this research contribute to the wider field of health communication or nursing research? A few sentences on the broader impact could strengthen the introduction | Addressed please see the last paragraph |
|  | it’s unclear how this study differs from previous research. Be more explicit about how this study fills the gap in the existing literature. | Addressed please see the last paragraph |
|  | • The role of nurses in the workshops isn’t clearly defined—were they participants, co-facilitators, or something else? | Addressed under the methods section |
|  | The role of researchers is vague. Clarify what exactly the researchers did—were they facilitators, analysts, or something else? | Described in each session |
|  | • The mention of the Culture-Centered Approach (CCA) is good, but it would be helpful to briefly explain what CCA is for readers unfamiliar with the term. | Addressed under CCA section |
|  | • The structure of the workshops needs more explanation— were there, and what was the format? | Addressed under **workshop organization and sessions** |
|  | • The process of thematic analysis needs more detail. How were the themes identified, and was there a process for ensuring reliability in the analysis? • The three themes are clear but lack some depth. How were these themes derived? Was there a specific process (e.g., coding, group discussions)? | Addressed under **data analysis section. A new section was added** |
|  | • Overall, the methodology seems appropriate, but more detail is needed to make it clearer, especially regarding the roles of participants, data collection, and ethical safeguards. | Thank you very much |
|  | • The procedure is not detailed enough in terms of how the workshops were conducted. For example, how were participants recruited and how were the workshops organized? • There’s no mention of how long each workshop lasted or how many total sessions were held. This is important to assess the depth and quality of the participatory process. • Facilitation process is unclear. Were the workshops led by the researchers, or were NICU nurses involved in facilitating some of the discussions? Clarify the role of the researchers and the nurses in the procedure. • It’s not clear if there was any follow-up with participants after the workshops. Did researchers engage with the nurses again to discuss the impact of the workshops or to follow up on outcomes? • The method of data collection (e.g., observations, notes, audio recordings) is not described. It’s important to explain how the data was gathered during the workshops and whether any tools were used (e.g., surveys or interview guides). | All of these were addressed in the procedure process |
|  | • The themes (nurses’ skills, communication policies, and shared understanding of diversity) are relevant, but the discussion could benefit from more exploration of how these themes specifically impact NICU practices or improve communication with parents. • There’s no discussion of any conflicting data or unexpected results. If there were any surprising findings or issues that emerged, it would be valuable to mention them and discuss how they were addressed. • The implications of the findings are mentioned, but the discussion could explore them in more detail. How exactly can these findings be applied to improve health communication in the NICU? What are the practical steps forward based on these results? There’s a need for a more detailed comparison between the results of this study and previous research. What does this study contribute that’s new or different? How do the findings compare to other studies • in the NICU or health communication field? | All of these were addressed in the discussion some sections were enhanced and new sections were added |
|  | • The conclusion is too vague. The statement about "producing significant changes" needs more detail—what exactly will change in NICU communication? | The conclusion was rewritten |
